# Police search and seizure during ongoing care in a pediatric emergency center in Korea: implications for medical training and the emergency care workforce

**DOI:** 10.3352/jeehp.2026.23.32

**Published:** 2026-10-16

**Authors:** Sun Huh

**Affiliations:** Institute of Medical Education, Hallym University College of Medicine, Chuncheon, Korea; The Catholic University of Korea, Korea

Adverse clinical outcomes, including death, profoundly affect patients, families, healthcare professionals, and trainees. Objective investigation and due legal process are necessary, and physicians are not exempt from the law. However, when unavoidable clinical events are not clearly distinguished from criminal conduct, or coercive investigations take place during active patient care, the effects may extend beyond those directly involved.

## Medicolegal risk as part of the hidden curriculum

Medical students and residents learn professional norms not only through formal curricula but also by observing how senior physicians are treated after adverse events and the personal risks of caring for high-risk patients. This informal learning constitutes the hidden curriculum [1]. Criminalization of medical practice and coercive investigations may become part of that curriculum, shaping perceptions of professional risk, specialty choice, and willingness to remain in high-risk fields.

In a self-selected online survey in Korea, concern about medical disputes and criminal liability was associated with defensive and avoidance-oriented practice among physicians [2]. Physicians in Korea may face criminal proceedings related to medical practice more frequently than their counterparts in some other countries. However, international comparisons should be interpreted cautiously because legal systems, case definitions, and data sources differ [3]. Among medical students and interns at one Korean institution, litigation risk and psychological burden were perceived as important barriers to choosing pediatrics [4].

This editorial considers how coercive investigations may affect trainees’ perceptions and resident recruitment into high-risk specialties. It proposes an alternative to addressing adverse medical outcomes primarily through criminal proceedings and outlines what medical students and residents should be taught in response.

## The police search and seizure at a university hospital

On September 30, 2026, a provincial police agency executed a search-and-seizure warrant at the pediatric emergency center of a university hospital in connection with the death of a child who had been transferred with cyanosis approximately 2 months earlier [5].

On October 1, 2026, the Korean Society of Pediatric Emergency Medicine issued the following statement about this coercive investigation [6]. The original Korean statement is available in Supplement 1.

Statement of the Korean Society of Pediatric Emergency Medicine on the search-and-seizure investigation conducted at a pediatric emergency center during ongoing patient care.
• We call for investigations that follow objective procedures for cooperation and safeguard the emergency care environment.
• Investigative practices that treat healthcare professionals as potential criminals solely based on an adverse outcome will undermine pediatric emergency care.

Recently, a child transferred to a pediatric emergency center with cyanosis died despite receiving treatment. The Society conveys its deepest condolences to the bereaved family following this tragic loss. We also wish to acknowledge the profound grief and sorrow experienced by the emergency medical staff involved.

However, the Society expresses serious concern and deep distress regarding the execution of a search-and-seizure warrant by investigative authorities on September 30, 2026 in a pediatric emergency center while active patient care was underway.

### Conducting a search-and-seizure investigation in an emergency department during active patient care threatens the safety of other pediatric patients.

A pediatric emergency center is a specialized clinical environment in which a delay of even seconds may affect a child’s survival. The unexpected arrival of investigative personnel and execution of a warrant inevitably distract healthcare professionals and disrupt care. Such a disruption may directly lead to treatment delays and safety incidents involving other critically ill pediatric patients awaiting emergency care.

### Investigative practices that treat best-effort emergency care as potentially unlawful will further weaken essential pediatric care.

In children with severe illness, the severity and rapid progression of disease can limit what medicine can achieve. If clinicians who undertake high-risk care in an effort to save lives are repeatedly treated as potential criminals merely because of an adverse outcome, there may soon be no healthcare professionals willing to sustain pediatric emergency care in Korea, where it is already under severe strain.

### In the era of electronic medical records, search-and-seizure investigations in emergency departments may violate the principle of proportionality.

Modern electronic medical records permanently retain logs of creation, modification, and access, often with timestamps precise to the second, making post hoc alteration or falsification fundamentally impossible. Materials required for an investigation can ordinarily be obtained through routine procedures and voluntary submission. Executing a search-and-seizure warrant against a hospital and healthcare professionals who have cooperated fully with an investigation constitutes an excessive measure that departs from the principles of proportionality and minimal intrusion.

Members of the Korean Society of Pediatric Emergency Medicine will always cooperate fully with transparent and objective efforts to establish the facts. However, investigative methods should be reconsidered if they compromise the ability to provide care in environments where children’s lives are at stake and discourage healthcare professionals from providing such care.

We strongly urge investigative and judicial authorities to establish sound standards for law enforcement that give the highest priority to patient safety and acknowledge the distinctive characteristics of emergency care environments.

October 1, 2026

Korean Society of Pediatric Emergency Medicine

## Medicolegal risk and specialty choice

Medicolegal risk can influence learning, clinical behavior, and career decisions. Most third-year US medical students in one study rarely worried about being sued, but 55.9% perceived that faculty were concerned about malpractice and 21.6% believed it would influence their specialty choice [7]. In a longitudinal study of 46 emergency medicine residents in California, concern about malpractice did not affect residency choice or markedly change decisions to perform procedures. However, it reduced interns’ enjoyment of medicine [8]. These findings do not establish that legal anxiety determines specialty choice, but they suggest that fear of liability shapes the learning environment.

A frequently cited international example is the case of Dr. Hadiza Bawa-Garba in the United Kingdom. As a pediatric specialty trainee, she was convicted of gross negligence manslaughter in 2015 after the death of a 6-year-old boy with pneumonia and sepsis under her care [9]. In 2018, on appeal by the General Medical Council, the High Court ordered her erasure from the medical register; the Court of Appeal later overturned that decision and restored the tribunal’s original sanction of suspension [10]. In a survey of 474 doctors in England conducted during this period, confidence in the fairness of the General Medical Council’s disciplinary procedures declined while the case was ongoing. Attitudes largely recovered afterward, however, and no measurable change in reflective practice or raising concerns was observed [11]. Among UK foundation doctors, the proportion proceeding directly into specialty training fell from 83% in 2010 to 38% in 2018, and the case was cited as one of several factors eroding junior doctors’ morale [12]. No study has shown that this case alone reduced applications to a particular specialty, but it illustrates how a single prosecution can shape trainees’ perceptions of professional risk.

Korea offers a more direct, though uncontrolled, example. After 4 neonates died at a university hospital in December 2017 and clinicians were arrested and later acquitted, the pediatric residency application rate reportedly fell from 112.1% in 2017 to 25.9% in 2023 [13]. This news report describes a temporal association; other factors, such as low reimbursement and the declining birth rate, likely contributed. However, the case may also have contributed to the low number of applicants to pediatrics.

Medical students in Korea reported that recent pediatric incidents affected their views of the specialty and expressed concern about litigation risks [4]. The question is whether such events shape the environment in which trainees make career decisions, rather than whether a single event changes specialty choice. Prior medicolegal litigation has also been associated with defensive medicine, including avoidance of high-risk patients and procedures [14], which is especially relevant to emergency and pediatric critical care.

## Toward an alternative to criminal handling of adverse medical outcomes

Concern about coercive investigations during active patient care now extends beyond the medical profession. On October 6, 2026, both the ruling and opposition parties called for investigative standards and manuals to prevent investigations from interfering with emergency care [15]. A spokesperson for the ruling Democratic Party stated that the facts of a medical accident must be thoroughly established, but that the investigation should not threaten the safety of other patients receiving care. The spokesperson pledged to work with the government, the medical community, and patient groups to improve investigative procedures and establish a rational system for resolving medical disputes. Members of the National Assembly Health and Welfare Committee from the opposition People Power Party, citing the hospital, stated that a critically ill 6-month-old infant was receiving emergency care at the bed subject to the search. They asked the police to disclose what measures had been taken to protect patients and called for practical standards covering consultation with medical staff, the scope and timing of searches, and protection of care pathways [15].

Beyond investigative standards, a more fundamental question remains. How long will the Korean government continue to address unwanted medical outcomes through criminal litigation, treating doctors as suspects? Without an adequate system within the profession to receive concerns from patients and medical institutions, investigate the facts, make judgments based on clinical expertise, and translate those judgments into education, supervision, and disciplinary action, medical disputes are likely to move directly to administrative or judicial bodies [16]. Although criminal justice is essential for intentional or serious criminal acts, relying on the criminal justice system to fill gaps in professional regulation is not a desirable approach to continuous improvement in patient safety. The Bawa-Garba case shows that an independent regulator alone does not prevent criminal prosecution [9,10]; clear thresholds for referring cases to criminal justice authorities are also needed. Thus, when unwanted outcomes occur after medical treatment, an independent medical regulatory authority that makes expert judgments and establishes a system to support the best possible subsequent care offers a way to ensure accountability based on expertise, improve patient safety, and reduce physicians’ unwarranted exposure to criminal proceedings.

## Implications for medical education

Training in medical law and dispute procedures is necessary but may be insufficient. Learners also need structured education in communication after adverse events, patient safety, and learning from errors [17], as well as decision-making under uncertainty, documentation, and balanced interpretation of legal risk. They should understand how professional self-regulation differs from criminal adjudication and which system will hold them accountable [16]. Institutions should support learners and clinicians involved in serious adverse events or medicolegal disputes. Healthcare students can themselves experience the second victim phenomenon, yet psychological support after adverse events remains fragmented [18].

## Conclusion

Students and residents observe how society and senior colleagues respond when clinicians accept responsibility for uncertain, high-risk care. Proportionate investigations that protect patient safety and a system for addressing adverse outcomes based on clinical expertise therefore matter both for legal fairness and for the educational environment in which future physicians choose their careers.

## Supplementary materials

Supplementary files are available from Harvard Dataverse: https://doi.org/10.7910/DVN/NFRIEA

Supplement 1. Original Korean statement by the Korean Society of Pediatric Emergency Medicine.
