# Supplementary material for: Police search and seizure during ongoing care in a pediatric emergency center in Korea: implications for medical training and the emergency care workforce
Source: J Educ Eval Health Prof. 2026 Oct 16;23:32. doi: 10.3352/jeehp.2026.23.32 (PMC13736233; doi:10.3352/jeehp.2026.23.32)
Supplement: Supplementary file 1 [file jeehp-23-32-suppl1.pdf]

진료 중인 소아응급센터 압수수색 수사에 대한소아응급의학회 성명서

- 객관적 협조 절차와 응급 진료 현장의 안전을 존중하는 수사를 촉구한다.

- 결과만을 근거로 의료진을 잠재적 범죄자 취급하는 수사는 소아응급을 고사시킨다.

대한소아응급의학회는 진료의 최전선인 응급실에서 열악한 환경 속에서도 소아청소년 환자의 생명을 지키기 위해 최선을 다하고 있습니다.

최근 청색증으로 소아응급센터에 내원해 치료를 받던 환아가 안타깝게 유명을 달리하는 사건이 발생했습니다. 본 학회는 먼저 안타깝게 목숨을 잃은 환아의 명복을 빌며, 슬픔을 겪고 계신 유가족분들께 위로의 말씀을 전합니다. 아울러 응급실 의료진 역시 무거운 슬픔과 비통한 마음을 안고 있음을 전합니다.

그러나 9 월 30 일 수사기관이 한창 진료가 이어지고 있던 소아응급센터 현장에 압수수색영장을 집행한 사태에 대해, 본 학회는 심각한 우려와 참담함을 표하지 않을 수 없습니다.

1. 진료 중인 응급실 내 압수수색 수사는 다른 환아들의 안전을 위협합니다.

소아응급센터는 수초의 지체로도 아이의 생사가 엇갈리는 특수 진료 구역입니다. 갑작스러운 수사 인력의 방문과 현장 집행 과정은 필연적으로 현장 의료진의 주의를 분산시키고 진료 흐름을 저해합니다. 이는 응급 처치를 기다리고 있는 또 다른 중증 응급 환아들의 치료 지연과 안전사고로 직결될 수 있는 매우 위험한 방식입니다.

2. 최선의 응급진료를 잠재적 위법으로 전제하는 수사 관행은 필수 소아의료를 위축시킵니다.

소아 중증 질환은 의료진이 총력을 다하더라도 질병의 진행 속도와 중증도로 인해 회복을 장담하기 어려운 의학적 한계가 존재합니다. 생명을 살리고자 위험을 무릅쓴 최선의 진료 결과를 두고 현장 의료진을 마치 잠재적 범죄자처럼 대하는 관행이 반복된다면, 이미 벼랑 끝에 서 있는 대한민국 소아응급의료의 현장을 지킬 의료진은 더 이상 남아있지 않을 것입니다.

3. 전자의무기록(EMR) 체계 하에서 응급실 압수수색 수사는 비례의 원칙에 어긋납니다.

현대 의료기관의 전자의무기록은 작성, 수정, 조회 이력이 초 단위 로그로 영구 기록되어 사후 위·변조가 원천적으로 불가능합니다. 수사에 필요한 자료는 통상적인 절차와 임의제출을 통해 객관적이고 충분하게 확보할 수 있습니다. 수사에 성실히 협조해 온 병원과 의료진을 상대로 굳이 압수수색영장을 앞세운 것은, 수사의 비례성과 최소 침해 원칙을 벗어난 과도한 조치입니다.

대한소아응급의학회 회원들은 투명하고 객관적인 사실 규명에 언제든 성실히 협조할 것입니다. 그러나 환아들의 생명을 다루는 현장의 진료권을 훼손하고 진료를 위축시키는 수사 방식은 재고되어야 마땅합니다.

수사기관과 사법기관은 응급 진료 현장의 특수성과 환자 안전을 최우선으로 고려하는 성숙한 법 집행 기준을 마련해 주실 것을 강력히 요청합니다.

2026 년 10 월 1 일

대 한 소 아 응 급 의 학 회
